# Risk Factors for Postpartum Hemorrhage and its Severe Forms with Blood Loss Evaluated Objectively – A Prospective Cohort Study

**DOI:** 10.1055/s-0040-1718439

**Published:** 2021-01-28

**Authors:** Anderson Borovac-Pinheiro, Filipe Moraes Ribeiro, Rodolfo Carvalho Pacagnella

**Affiliations:** 1 Department of Obstetrics and Gynecology, Universidade Estadual de Campinas, Campinas, SP, Brazil

**Keywords:** risk factors, postpartum hemorrhage, maternal mortality, fatores de risco, hemorragia pós-parto, mortalidade materna

## Abstract

**Objective** To identify risk factors related to postpartum hemorrhage (PPH) and severe PPH with blood loss quantified objectively.

**Methods** This is a complementary analysis of a prospective cohort study that included pregnant women delivering vaginally. The total blood loss was obtained through the sum of the volume collected from the drape with the weight of gauzes, compresses and pads used by women within 2 hours. Exploratory data analysis was performed to assess mean, standard deviation (SD), frequency, percentage and percentiles. The risk factors for postpartum bleeding were evaluated using linear and logistic regression.

**Results** We included 270 women. The mean blood loss at 120 minutes was 427.49 mL (±335.57 mL). Thirty-one percent (84 women) bled > 500 mL and 8.2% (22 women) bled > 1,000 mL within 2 hours. Episiotomy, longer second stage of labor and forceps delivery were related to blood loss > 500 mL within 2 hours, in the univariate analysis. In the multivariate analysis, only forceps remained associated with bleeding > 500 mL within 2 hours (odds ratio [OR] = 9.5 [2.85–31.53]). Previous anemia and episiotomy were also related to blood loss > 1,000mL.

**Conclusion** Prolonged second stage of labor, forceps and episiotomy are related to increased incidence of PPH, and should be used as an alert for the delivery assistants for early recognition and prompt treatment for PPH.

## Resumo

**Objetivo** Identificar os fatores de risco para hemorragia pós-parto e hemorragia pós-parto grave com o sangramento pós-parto avaliado objetivamente.

**Métodos** Trata-se de uma análise complementar de um estudo de coorte prospectivo que incluiu somente mulheres que evoluíram para parto vaginal. O total de perda sanguínea foi avaliado objetivamente durante 24 horas pós-parto através da soma da quantidade de sangue mensurada através de um coletor de sangue pós-parto somado ao peso de compressas, gases e absorventes utilizados no período pós-parto. Análises exploratórias dos dados foram realizadas através do cálculo de médias, desvio-padrão (DP), frequência, porcentagem e percentis. Os fatores de risco foram avaliados através de regressão linear e logística.

**Resultados** Foram incluídas 270 mulheres. A média de perda sanguínea pós-parto após 120 minutos foi de 427.49 mL (±335.57 mL). Trinta e um por cento (84 mulheres) sangraram > 500 mL e 8,2% (22 mulheres) sangraram > 1.000 mL em 2 horas. Episiotomia, segundo período do parto prolongado e uso de fórceps estiveram associados a perda sanguínea > 500 mL em 2 horas. Na análise multivariada, somente fórceps manteve-se entre os fatores de risco para sangramentos superiores a 500 mL em 2 horas ( *odds ratio* [OR] = 9.5 [2.85–31.53]). Anemia prévia e episiotomia estiveram associadas com perda sanguínea > 1.000 mL.

**Conclusão** Segundo período do parto prolongado, fórceps e episiotomia estão associados a aumento da incidência de hemorragia pós-parto e devem ser usados como um alerta para os profissionais de saúde para o reconhecimento precoce e tratamento imediato da patologia.

## Introduction

In spite of the efforts to decrease maternal mortality worldwide, every day 800 women die due to complications related to pregnancy and childbirth. 1 Behind the numbers, these premature deaths lead to an impact on families, societies and economies and basically, for the children, mean the loss of a caregiver and nurturing figure. 2 At least for the past 25 years, maternal hemorrhage remains the leading cause of maternal mortality worldwide and the majority of deaths occur at the postpartum period in low sociodemographic index countries. 3 4

Postpartum hemorrhage (PPH) is defined by the World Health Organization (WHO) as bleeding > 500 mL within 24 hours after delivery and severe PPH as bleeding > 1,000 mL during the same period. 5

For the last years, PPH and severe PPH is increasing around the world, even in developed countries. 6 7 To recognize women at risk who could potentially develop PPH is the first action to prompt treatment to avoid deaths and near-misses due to PPH.

Nevertheless, several studies have shown conflicting risk factors for PPH based on visual estimation of blood loss. 6 7 8 9 10 While some studies identified age < 20 years old, hypertension and multiple gestations 6 8 as a risk factor for PPH, others did not find the relationship among these potential risk factors and postpartum bleeding. 7 9 11 The fact is that the majority of the studies evaluate PPH using a visual estimative of blood loss, a low accuracy method to measure postpartum bleeding. 12 13 14

The present study aimed to identify risk factors related to PPH and severe PPH with blood loss quantified objectively.

## Methods

This is a complementary analysis of a prospective cohort study designed to identify if shock index and other vital signs could be useful to predict PPH (not published). It included pregnant women with gestational age > 34 weeks delivering vaginally at the Women's Hospital (Hospital da Mulher J.A Pinotti, Campinas, São Paulo, Brazil) between 1 February 2015 and 31 March 2016. The exclusion criteria were the presence of one or more of these conditions: gestational age < 34 weeks, hypertension, hypo or hyperthyroidism without treatment, coagulopathy, antepartum hemorrhage, any cardiac disease and infections with fever or sepsis.

During the labor, at the obstetric ward, women were invited to participate in the study, and if accepted, they signed an informed consent form. A data collection form was filled with information from the women's interview added with information from the medical records. The hemoglobin level was checked in prenatal records. If the last dosage had been made before 3 months, we collected a new blood count before delivery. Previous anemia was defined as hemoglobin levels < 11 g/dL. If the women progressed to C-section they were excluded from the study.

**Fig. 1:**
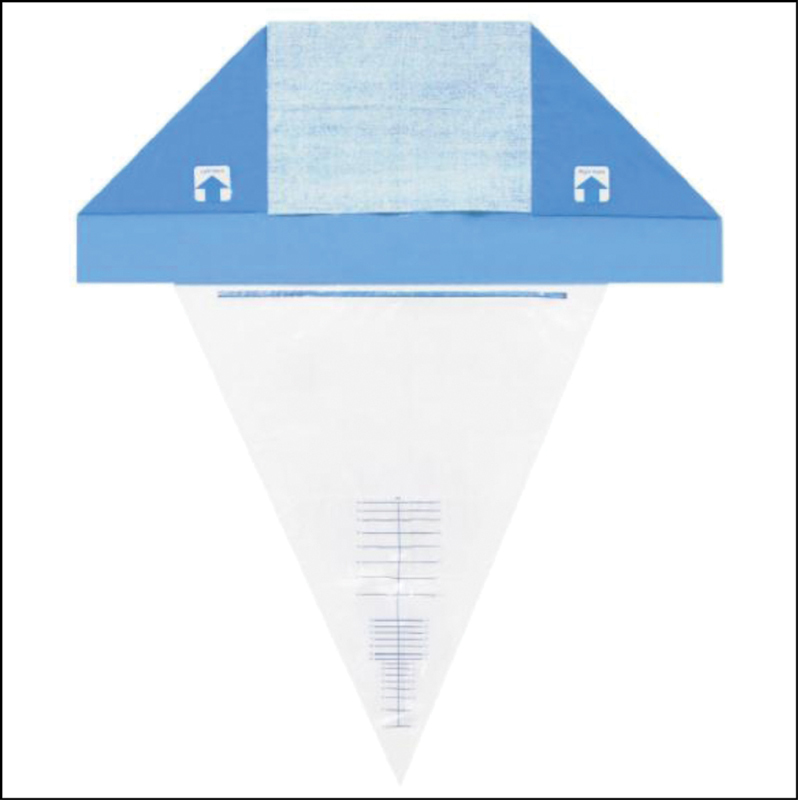
Calibrated drape used to measure objectively blood loss after fetal delivery (BRASSS_V drape_Maternova_Providence, RI, USA).

Immediately after the fetal delivery, trained research assistants placed a calibrated drape under the women's buttocks (BRASSS_V drape_Maternova_Providence, RI, USA – Fig. 1 ). The total blood loss was obtained through the sum of the volume collected from the drape with the weight of gauzes, compresses and pads (subtracting the dry weight) used by women within 2 hours. For volume estimation, we considered the density of blood to be 1 g/mL. 15

For the statistical analyses, we identified the possible risk factors that could be related to PPH and severe PPH. Therefore, we performed exploratory data analysis to assess mean, standard deviation (SD), minimum, median, maximum, frequency, percentage and percentiles. The risk factors for postpartum bleeding were evaluated using linear and logistic regression. All statistical analysis was made using SAS 9.4 (SAS Institute Inc., SAS São Paulo, São Paulo, Brazil) and we defined a significance level of 5%.

The Institutional Review Board (IRB of the Universidade de Campinas, Campinas, SP, Brazil) approved the main study, which included evaluating the risk factors for PPH (CAEE: 26787114.3.0000.5404). Without any participation in planning, designing, implementing, collecting data, analysis and interpreting results, Centro de Pesquisas em Saúde Reprodutiva de Campinas (CEMICAMP) and Fund for Support to Teaching, Research and Outreach Activities (Faepex-UNICAMP) supported the study.

## Results

From the 319 eligible women, 8 denied participation and 41 progressed to C-section. Therefore, we included 270 women. The mean blood loss at 120 minutes was 427.49 mL (±335.57 mL). Thirty-one percent (84 women) of the sample bled > 500 mL and 8.2% (22 women) bled > 1,000mL within 2 hours. On the other hand, among those who bled less, 93 women (34.4%) had blood loss ≤ 300 mL and 125 (46.3%) had blood loss ≤ 400mL, below the mean blood loss. Forty-seven women (17.4%) arrived at the hospital during the second stage of labor. Sociodemographic and obstetrics characteristics are shown in Table 1 .

**Table 1:** Sociodemographic and Obstetrics characteristics

Characteristics	n	Mean ± SD
Age (years old)	270	24.67 ± 6.2
BMI (antepartum) ^a^	244	28.85 ± 4.6
Parity	270	0.80 ± 1.10
Gestational age (in weeks)	270	38.93 ± 1.47
Education (in years) ^b^	231	9.91 ± 2.5
Time to second stage (in minutes) ^c^	223	32.47 ± 34.7
Initial Hb (in g/dL) ^d^	260	11.45 ± 0.1
Ethnicity - white ^e^	178 (67%)	
Previous C-Section	42 (15.5%)	
Spontaneous onset of labor	203 (75.2%)	
Anesthesia/analgesia (yes)	170 (63%)	
Mode of delivery		
vaginal	247 (91.5%)	
forceps	23 (8.5%)	
Episiotomy (yes)	96 (36%)	
Laceration (≥ grade 2)	155 (57.1%)	
Blood loss within 120 minutes		
≥ 500 mL	84 (31%)	
≥ 1000 mL	22 (8.2%)	

Abbreviations: BMI, body mass index; Hb, hemoglobin.

Missing: ^a^ 26; ^b^ 39; ^c^ 47; ^d^ 10; ^e^ 7; ^a^ in Kg/m ^2^ .

No women in our sample had intensive care unit (ICU) admission or surgical procedures. Only four women received blood transfusions due to PPH. Among those who bled > 500 mL, 18 women (21.4%) had forceps delivery and 38 (45.2%) had an episiotomy. And among those who bled > 1,000 mL, 4 women (18.2%) had forceps delivery, 11 (50%) had an episiotomy and 6 (27.3%) had previous anemia. The logistic regression to evaluate factors related to blood loss after delivery is shown in Table 2 .

**Table 2:** Univariate and multivariate analysis of risk factors to blood loss ≥ 500mL and ≥ 1000mL in 2 hours

		500 mL within 2 hours		1,000 mL within 2 hours	
	n	OR	*p-value*	OR	*p-value*
		(95%CI)		(95%CI)	
**Univariate analysis**					
Age					
≤ 19 years old	63	1.51	0.172	1.60	0.329
		(0.83 - 2.71)		(0.62 - 4.11)	
20–35 years old	191	Ref.			
≥ 35 years old	16	1.00	0.990	1.67	0.516
		(0.34 - 2.99)		(0.35 - 7.87)	
Ethnicity					
white	178	Ref.			
non-white	85	0.83	0.534	2.24	0.085
		(0.47 - 1.47)		(0.89 - 5.61)	
Schooling (mean in years)	231	1.04	0.431	0.97	0.813
		(0.93 - 1.17)		(0.80 - 1.18)	
Overweight (BMI ^a^ ≥25)	104	1.36	0.272	0.94	0.900
		(0.78 - 2.36)		(0.34 - 2.55)	
Obesity (BMI ^a^ ≥ 30)	89	1.03	0.913	1.60	0.350
		(0.58 - 1.82)		(0.59 - 4.31)	
Multiparity (two or more previous deliveries)	53	0.65	0.227	0.87	0.817
		(0.32 - 1.30)		(0.28 - 2.70)	
Gestational age					
34–40 weeks	146	0.92	0.764	1.64	0.277
		(0.53 - 1.59)		(0.67 - 4.03)	
40 weeks	102	Ref.			
Previous C-section	42	1.13	0.731	1.65	0.351
		(0.56 - 2.28)		(0.57 - 4.75)	
Anemia (hemoglobin ≤ 11 g/dl)	43	1.60	0.175	2.82	0.037
		(0.81 - 3.15)		(1.06 - 7.47)	
Spontaneous labor	203	0.58	0.062	0.87	0.780
		(0.32 - 1.03)		(0.32 - 2.32)	
Duration of second-stage of labor ≥ 30 minutes	223	1.88	0.032	1.90	0.230
		(1.05–3.37)		(0.66–5.45)	
Anesthesia (yes)	168	1.225	0.458	1.068	0.886
		(0.71–2.09)		(0.43–2.64)	
Episiotomy (yes)	96	2.39	0.001	3.05	0.017
		(1.39 - 4.10)		(1.12 - 7.66)	
Laceration (≥ grade 2)	144	1.03	0.924	1.64	0.300
		(0.60 - 1.74)		(0.64 - 4.22)	
Forceps (yes)	23	9.87	<0.001	2.68	0.101
		(3.53 - 27.65)		(0.82 - 8.72)	
Multivariate analysis					
Forceps (yes)	260	9.48	<0.001		
		(2.85 - 31.53)			
Duration of second stage of labor ≥ 30 minutes	260	1.05	0.883		
		(0.57–2.10)			
Episiotomy (yes)	260	1.49	0.25		
		(0.75–2.96)			

Abbreviations: BMI, body mass index; CI, confidence interval; OR, odds ratio.

Episiotomy, longer second stage of labor and forceps delivery were related to blood loss > 500mL within 2 hours. The multiple analysis ( *n* = 260) shows that forceps delivery had an odds ratio (OR) of 9.48 (95% confidence interval [CI]: 2.85–31.53) for bleeding > 500 mL within 2 hours.

Previous anemia, longer second stage of labor and episiotomy were also related to blood loss > 1,000 mL. Nevertheless, the multiple analyses had not shown a risk factor related to bleeding > 1,000mL within 2 hours after delivery.

## Discussion

Our study aimed to evaluate risk factors for PPH and severe PPH within 2 hours after delivery with blood loss quantified objectively. Episiotomy, forceps and longer second stage of delivery were related to PPH, and episiotomy and previous anemia were related with severe PPH.

The actual research related to PPH is concerned with the early identification of PPH in an attempt to promote, with the prompt and accurate treatment, the decrease of maternal mortality and near-miss due to PPH. The identification of risk factors could contribute as an adjunct for early recognition of PPH. 10 16 17

The main contributors to developing PPH and severe PPH in our study were forceps, longer second stage of labor and episiotomy, which are frequently described in the literature; nevertheless, in our study, maternal age > 35 years, multiparity, induced labor and previous C-sections were not related to PPH and severe PPH, as they were found as risk factors for PPH in other several studies. 6 7 8 9 18

Forceps delivery had an OR of 9.48 for the risk of developing PPH, although our analysis does not show forceps as a risk factor for severe PPH as demonstrated in other studies. 8 9 Perhaps, this difference could be explained by the quantifying method to measured postpartum bleeding or by the number of women with severe PPH in our study, as only 22 women had severe PPH, while other studies, which were population studies, found > 3,000 women with severe PPH. 8 9

Previous anemia (hemoglobin level < 11 g/dl) was found to be a risk factor for severe PPH. This is in agreement with another study that found hemoglobin levels < 9 g/dl as a risk factor for severe PPH, OR = 2.20 (1.63–3.15). 9 Our data shows the importance of adequate antenatal care with diagnosis and treatment of anemia as a changeable risk factor for PPH. Iron supplementation is a recommendation of the WHO during pregnancy and the postpartum period 19 20 and could decrease a part of the incidence of PPH. Also, the presence of previous anemia may influence the recovery after bleeding.

Comparing the objective method of measuring PPH, one study from Uganda assessed postpartum bleeding using a calibrated drape. The risk factors found by them were HIV positive, multiple pregnancy and macrosomia. 11 Nevertheless, they had a very low frequency of PPH (9%) and severe PPH (1.2%) 11 compared with our data, which shows respectively 31% and 8.2% of frequency. In our sample, only 11 women delivered babies > 4,000 g and 8 of them had postpartum bleeding > 500mL.

Our data showed that prolonged second-stage labor, forceps and episiotomy, which are very linked to each other, are related to an increased incidence of PPH. In the modern assistance to labor and delivery, it is recommended to respect the obstetrical physiological variations found among women. 21 However, if an operative delivery or even episiotomy is required, the team should be prepared for the possibility of facing a PPH, and these three risk factors should be used as an alert for the delivery assistants for early recognition and prompt treatment for PPH.

Although our study has the strength of evaluating objectively the postpartum bleeding for 2 hours, a rare characteristic found in studies that evaluate risk factors, it has some limitations. Our sample size is limited, only 270 women were included, and we excluded pregnant women with hypertension, a known risk factor for PPH. Ideally, we should have divided the comparison of the second stage of labor between above and below 1 hour. However, our sample size was not sufficient for this. Future research with PPH should be done evaluating postpartum bleeding objectively in a large sample size and with no exclusion criteria.

## Conclusion

Prolonged second stage of labor, forceps and episiotomy are related to PPH, and should be used as an alert for the delivery assistants for early recognition and prompt treatment for PPH.
